# The High North

**Published:** 1884-05

**Authors:** 


### THE HIGH NORTH.

IT Sounds Strange to hear of a steamboat on the South Saskatchewan river in Manitoba, Canada; the Saskatchewan with its north and south branches, rises in the Rocky mountains and runs east into Lake Winnipeg. As there are no settlements on its banks, and the country is described as fertile and tillable, it would seem that the northern limit of profitable farming has not yet been reached in that vast region once regarded as a vast desolation, fit only for fur-bearing animals Not far north of the Saskatchewan the Arctic system of rivers has its head waters—the Athabaska and Peace, which unite to form the Mackenzie, the mighty stream which discharges its waters into the Polar sea above the Arctic circle. Here is a vast region almost as large as the United States, abounding in rivers and lakes, some of which are known to be navigable. The lower part of it will in a few years, be provided with railroads; and we may yet live to see the foundations of an empire laid in its unbroken solitudes.

